# Integration of regulatory signals through involvement of multiple global regulators: control of the *Escherichia coli gltBDF *operon by Lrp, IHF, Crp, and ArgR

**DOI:** 10.1186/1471-2180-7-2

**Published:** 2007-01-18

**Authors:** Ligi Paul, Pankaj K Mishra, Robert M Blumenthal, Rowena G Matthews

**Affiliations:** 1 Life Sciences Institute, University of Michigan, Ann Arbor, Michigan 48109, USA; 2 Department of Medical Microbiology and Immunology, University of Toledo Health Science Campus, Toledo, Ohio 43614-5806, USA; 3 Program in Bioinformatics and Proteomics/Genomics, University of Toledo Health Science Campus, Toledo, Ohio 43614-5806, USA; 4 Biophysics Research Division, University of Michigan, Ann Arbor, Michigan 48109, USA; 5 Department of Biological Chemistry, University of Michigan, Ann Arbor, Michigan 48109, USA; 6 Vitamin Metabolism and Aging Laboratory, Jean Mayer USDA Human Nutrition Research Center on Aging, Tufts University, Boston, Massachusetts 02111, USA

## Abstract

**Background:**

The glutamate synthase operon (*gltBDF*) contributes to one of the two main pathways of ammonia assimilation in *Escherichia coli*. Of the seven most-global regulators, together affecting expression of about half of all *E. coli *genes, two were previously shown to exert direct, positive control on *gltBDF *transcription: Lrp and IHF. The involvement of Lrp is unusual in two respects: first, it is insensitive to the usual coregulator leucine, and second, Lrp binds more than 150 bp upstream of the transcription starting point. There was indirect evidence for involvement of a third global regulator, Crp. Given the physiological importance of *gltBDF*, and the potential opportunity to learn about integration of global regulatory signals, a combination of *in vivo *and *in vitro *approaches was used to investigate the involvement of additional regulatory proteins, and to determine their relative binding positions and potential interactions with one another and with RNA polymerase (RNAP).

**Results:**

Crp and a more local regulator, ArgR, directly control *gltBDF *transcription, both acting negatively. Crp-cAMP binds a sequence centered at -65.5 relative to the transcript start. Mutation of conserved nucleotides in the Crp binding site abolishes the Crp-dependent repression. ArgR also binds to the *gltBDF *promoter region, upstream of the Lrp binding sites, and decreases transcription. RNAP only yields a defined DNAse I footprint under two tested conditions: in the presence of both Lrp and IHF, or in the presence of Crp-cAMP. The DNAse I footprint of RNAP in the presence of Lrp and IHF is altered by ArgR.

**Conclusion:**

The involvement of nearly half of *E. coli*'s most-global regulatory proteins in the control of *gltBDF *transcription is striking, but seems consistent with the central metabolic role of this operon. Determining the mechanisms of activation and repression for *gltBDF *was beyond the scope of this study. However the results are consistent with a model in which IHF bends the DNA to allow stabilizing contacts between Lrp and RNAP, ArgR interferes with such contacts, and Crp introduces an interfering bend in the DNA and/or stabilizes RNAP in a poised but inactive state.

## Background

A small number of global regulatory proteins appear to play a central role in integrating the regulatory architecture of Escherichia coli, so as to promote coherent transcriptional responses to environmental changes. Just seven proteins (ArcA, Crp, FIS, Fnr, IHF, Lrp, and NarL) directly control expression of about half of all genes in E. coli [1]. We report here direct evidence that three of these seven proteins, plus a more specific regulator, cooperate to control an operon critical to nitrogen metabolism.

**Figure 1:**
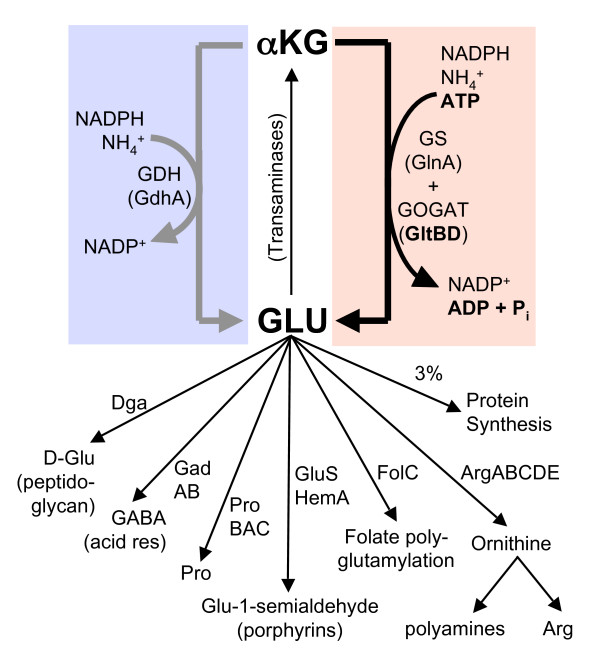
**Biosynthesis and metabolic uses of L-glutamic acid**. The two alternative pathways from α-ketoglutarate (αKG) to glutamate (Glu) are shown by the thick arrows, as described by equations 1–4 in the Introduction. The gray arrow (left) represents equation 1, while the black arrow (right) represents the net sum of equations 2 and 3 (*i.e.*, equation 4). Glu is one of the small minority of amino acids for which the great bulk of the molecules are used for purposes other than direct incorporation into proteins [2]. A number of transaminases use the α-amino group of Glu. Other uses of Glu were identified from the BioCyc website [63].

There are two main pathways for assimilating ammonia into glutamate in Escherichia coli [2] (see Fig. 1). In the presence of high ammonia concentrations and limited carbon/energy, glutamate dehydrogenase (GdhA) produces glutamate from ammonia, α-ketoglutarate, and NADPH [equation 1, and left side of Fig. 1].

NH_4_^+ ^+ α-Ketoglutarate + NADPH → Glutamate + NADP^+ ^    [1]

At low ammonia concentrations, a two-enzyme cycle is used instead. First, glutamine synthetase (GlnA) produces glutamine from ammonia, glutamate and ATP [equation 2]. Then glutamate synthase (GltBD) produces two molecules of glutamate from glutamine, α-ketoglutarate, and NADPH [equation 3], with one of the glutamate molecules going back into the cycle and the other representing net ammonia incorporation [equation 4, and right side of Fig. 1]. The only difference between equations [1] and [4] is the involvement of ATP.

NH_4_^+ ^+ Glutamate + ATP → Glutamine + ADP + P_i _    [2]

Glutamine + α-Ketoglutarate + NADPH → 2 Glutamate + NADP^+ ^    [3]

NH_4_^+ ^+ α-Ketoglutarate + NADPH + ATP → Glutamate + NADP^+ ^+ ADP + P_i_   [4]

In the presence of high ammonia levels, GltBD activity would waste ATP as a result of unnecessary glutamine turnover. The pathway represented by equation 4 is estimated to account for a remarkable 15% of total ATP turnover during growth in glucose minimal medium [2], so the ATP wastage would be substantial. However, insufficient GltBD activity in the face of dropping ammonia levels would lead to cessation of growth [3,4] and a competitive disadvantage. Clearly the level of GltBD must be very carefully controlled so as to anticipate the probable near-term needs of the cell.

Our interest in gltBDF, the operon that includes structural genes for GltBD, stems from its membership in the regulon controlled by Lrp (leucine-responsive regulatory protein) [5]. There is no obvious single role for Lrp, but three broad themes stand out. First, Lrp appears to sense the shift between two major E. coli environments, "the gut and the gutter" [6], activating many amino acid biosynthetic operons (such as gltBDF) and repressing many catabolic ones. Second, and partially overlapping the first, Lrp plays an important role in regulating nitrogen metabolism [2,5,7]. Third, Lrp appears to play an important role in preparing the cell for stationary phase [6,8-13].

**Figure 2:**
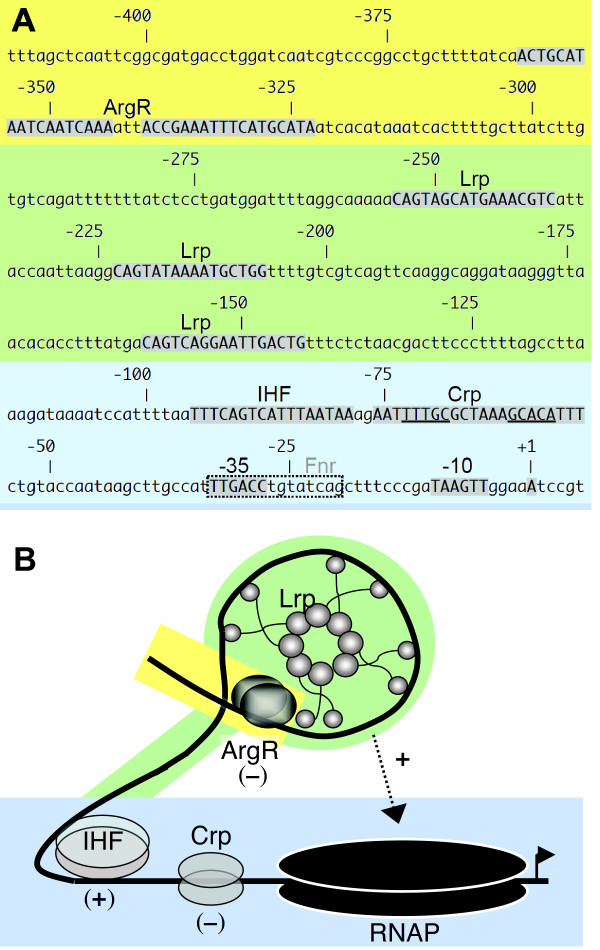
***gltBDF *promoter region**. **A. **Sequence of region upstream of *gltB*. Nucleotides shown as shaded capitals represent promoter elements or binding sites for the indicated regulatory proteins, as identified in this work and earlier studies (see references in text). The borders shown for binding sites are based on comparison to consensus sequences, and are consistent with DNAse I footprints reported in this study. The binding site for Fnr is shown as a dashed box because it has not been experimentally confirmed (see information on *gltBDF *at BioCyc.org). The color-shaded background indicates three broad regions of the promoter (initiation region, blue; Lrp-binding region, green; ArgR-binding region, yellow). **B**. A model for *gltBDF *transcriptional regulation. Lrp and IHF bind and, according to the model, bend *gltBDF *promoter DNA such that an upstream regulatory sequence or Lrp surface required for stable binding of RNAP is brought in contact with RNAP that is loosely associated with the promoter region. This positive interaction (and not Lrp binding) is partially abolished by ArgR-Arg bound to *glt *DNA. In the presence of Crp-cAMP, RNAP association with the promoter is stabilized but in a nonproductive manner, leading to transcriptional repression. Background shading matches that in part A.

In the *gltBDF *operon, IHF binds the region from -75 to -113, while the three Lrp binding sites are centered at nucleotides -152, -215 and -246 relative to the transcription start site [5,14] (Fig. 2A). This arrangement leaves space for other proteins to bind, and it seemed likely that additional regulators would be involved in controlling a pathway that can account for 15% of the cell's ATP flux. There is some evidence for effects (direct or indirect) of other regulators on *gltBDF *expression, including Crp [15], Fnr [16], Nac [17], and GadE [18] (see information on the *gltBDF *operon at EcoCyc.org [19]). We report here that *gltBDF *is directly and negatively controlled by Crp and ArgR, in addition to the direct positive control by Lrp and IHF. We also show that RNAP binds stably to the *gltBDF *promoter in the presence of Lrp and IHF, or in the presence of Crp-cAMP.

## Results

### Effects of Lrp and IHF on RNAP binding to the *gltBDF *promoter region

**Figure 3:**
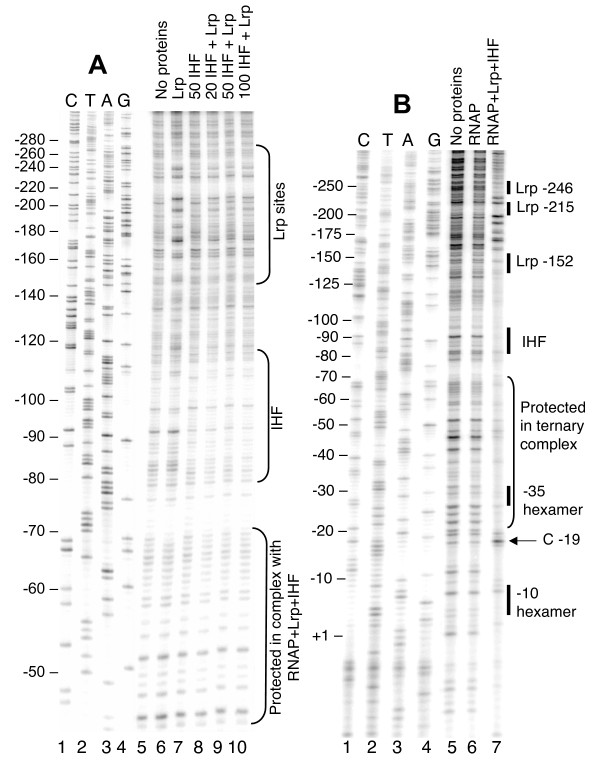
**DNAse I protection of the *gltBDF *promoter region by Lrp, IHF, and RNA polymerase**. **A**. DNase I protection assay of *gltBDF *promoter region in the presence of both Lrp and IHF. A fragment of *gltB *DNA from -325 to +8 was used in the reaction. The first 4 lanes represent a sequencing ladder of the region. In all cases where it was added, Lrp was used at 2 nM. The amounts of IHF are shown in nM. **B**. A fragment of *gltB *DNA from -571 to +51, labeled at the +51 end of the template strand using ^32^P, was used. When present, the concentration of RNAP was 30 nM, Lrp 100 nM and IHF 100 nM. The first 4 lanes represent a sequencing ladder of the template strand of the region.

We have previously shown that Lrp and IHF bind simultaneously to *gltBDF *promoter (P*gltB*) DNA, yielding a band with intermediate gel shift mobility between the IHF-DNA and Lrp-DNA complexes (Fig. 3C in [14]). Fig. 3A shows that the Lrp-binding region yields a subtly altered Lrp-dependent DNAse I footprint in the presence of IHF, though the IHF footprint is not obviously changed by the presence of Lrp.

RNA polymerase holoenzyme (RNAP) binds to P*gltB *in the absence of other proteins, as revealed by electrophoretic mobility shift of a DNA segment from -406 to +131 (relative to the transcription start; not shown). However DNAse I footprinting analysis of this binary complex revealed no obvious regions of protection (Fig. 3B, compare lanes 5 and 6). Since the absence of either Lrp or IHF leads to a >30-fold reduction in *gltBDF *transcription [5,14], we investigated whether these two proteins influence RNAP binding to P*gltB*. Mobility shift experiments were not informative as, under our conditions, the large shift due to RNAP alone was indistinguishable from shifts due to RNAP together with Lrp and/or IHF. However DNAse I protection analysis revealed that the combination of Lrp, IHF and RNAP results in a footprint in the promoter region, with strongly enhanced cleavage at the -19 position of the template strand and protection in the remainder of the region between the -10 and -35 hexamers (Fig. 3B, compare lane 7 to lanes 5 and 6). This protection extended ~30 bp upstream of the -35 hexamer, merging with the footprint of IHF, and some protection was visible even farther upstream between the IHF and Lrp binding sites. A combination of just Lrp and IHF does not result in any additional footprint aside from those of the individual proteins [14], and in particular the extended protection upstream of the -35 hexamer is not seen in the absence of RNAP (Fig. 3A).

### Crp-cAMP regulation of *gltBDF *transcription

**Figure 4:**
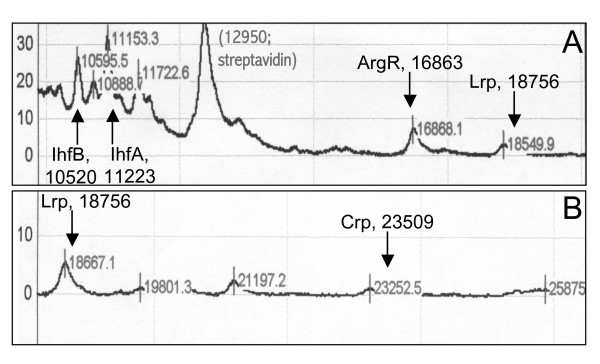
**Masses of polypeptides associated with *gltB *DNA *in vitro***. Surface-enhanced laser desorption and ionization (SELDI; Ciphergen Biosystems) was used to identify proteins from whole-cell *E. coli *extracts binding to biotin-labeled *gltB *DNA (from -406 to +246). The DNA was attached to streptavidin-coated chips, incubated with the extracts, and washed prior to mass analysis. Parts A and B show different mass ranges. The vertical arrows indicate the theoretical positions of the indicated polypeptides; in each case the mass shown assumes removal of the N-terminal methionine.

We searched for proteins from an *E. coli *whole-cell extract that bound to immobilized, biotinylated P*gltB *DNA, using Ciphergen ProteinChips™ and mass spectrometry. This method had previously implicated IHF as a factor involved in *gltBDF *regulation, as we subsequently confirmed [14], but additional mass peaks representing other DNA binding proteins had not yet been investigated. The analysis suggested ArgR (regulator for the arginine regulon) and Crp (catabolite repressor protein) as potential additional regulators of *gltBDF *(Fig. 4). Several decades ago Prusiner *et al*. reported negative regulation of glutamate synthase expression by Crp [15]. Specifically, glutamate synthase activity is doubled in a mutant lacking adenylate cyclase (*cya*), and reduced 1.5-fold by addition of exogenous cAMP or growth with glycerol as the carbon source (which increases cAMP levels) [15]. However, it was not determined whether these results involved direct interactions between Crp and P*gltB*.

**Figure 5:**
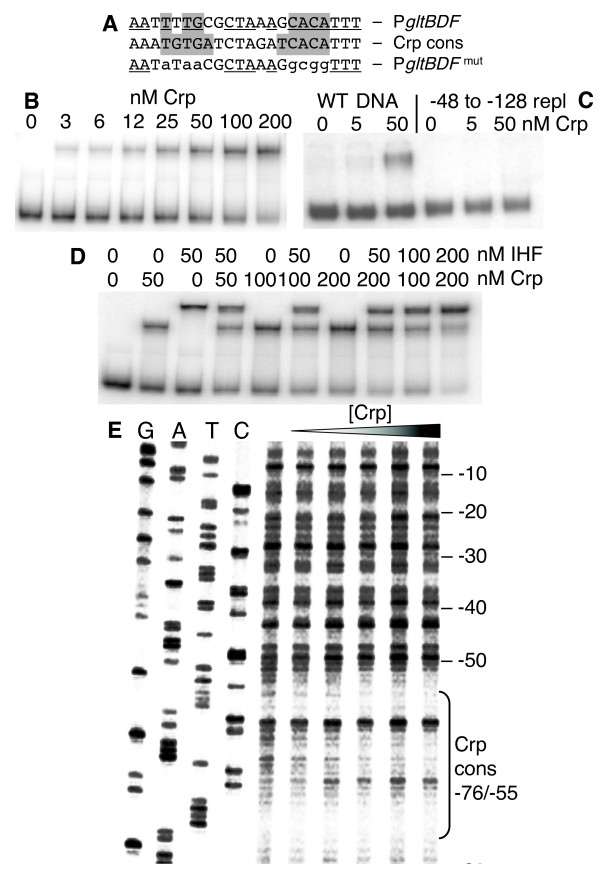
**Interactions between Crp and the *gltBDF *promoter region**. **A**. The *glt *Crp-binding sequence (top) compared to the consensus Crp binding sequence (middle). The most-conserved elements of the Crp sequence are shaded, and matches to the consensus are underlined. The bottom sequence shows the mutations (lowercase) that were introduced for the experiment shown in Fig. 6. **B**. Gel mobility shift analysis of Crp binding to *glt *DNA region -406 to +246. The running buffer and gel contained 20 μM cAMP. **C**. Gel mobility shift analysis as in (B), except that two DNAs are used. One is WT, and the other has the region from -128 to -48 replaced by an equal length of exogenous DNA (amplified from the coding region of the *cat *gene).** D**. Gel mobility shift assay showing simultaneous binding of Crp and IHF to *gltBDF *DNA. A fragment of *gltB *DNA from -203 to +8 was used in the assay. **E**. DNAse I protection of *glt *DNA by Crp binding. A *gltB *DNA fragment from -203 to +161 labeled at the -203 end of the partner strand was used for the assay. The first 4 lanes represent the sequencing ladder of the partner strand. Crp concentrations of 0, 12.5, 25, 50, 100 and 200 nM were used in the assay.

Our inspection of the *gltBDF *DNA sequence revealed a potential Crp-binding site between -76 and -55 (Fig. 5A, top two lines). We have not attempted to measure transcription of a *gltB-lacZ *fusion in isogenic *crp *and *crp*^+ ^strains, to complement the earlier data of Prusiner et al. [15]. This is because the *crp *mutant grows much more slowly than its isogenic partner [20], and comparisons would be complicated by the different growth rates [21]. However we have taken three complementary approaches to demonstrate a direct effect of Crp-cAMP on *glt *transcription.

First, we carried out mobility shift assays using purified Crp and a *gltBDF *DNA fragment containing the region from -406 to +246. Crp bound the *glt *DNA with an apparent K_d _of 35 nM in the presence of 20 μM cAMP (Fig. 5B). Replacing the region between -121 and -48 with heterologous DNA [14] resulted in loss of detectable binding (Fig. 5C). When cAMP was not added to the gel, but was present in the binding reaction, binding was still observed but the shifted bands were smeared, suggesting that the complexes were unstable under this condition (not shown).

Second, we carried out DNAse I footprint analyses, using purified Crp, cAMP, and P*gltB *DNA. A defined region of partial protection was seen between -71 and -52 on the non-template strand (Fig. 5E), and -72 to -57 on the template strand (not shown). Thus Crp-cAMP interacts directly with P*gltB*. The Crp binding is centered at -65.5, in the predicted binding sequence (-76 to -55), and between the -35 hexamer and the IHF binding site (Fig. 2A).

**Figure 6:**
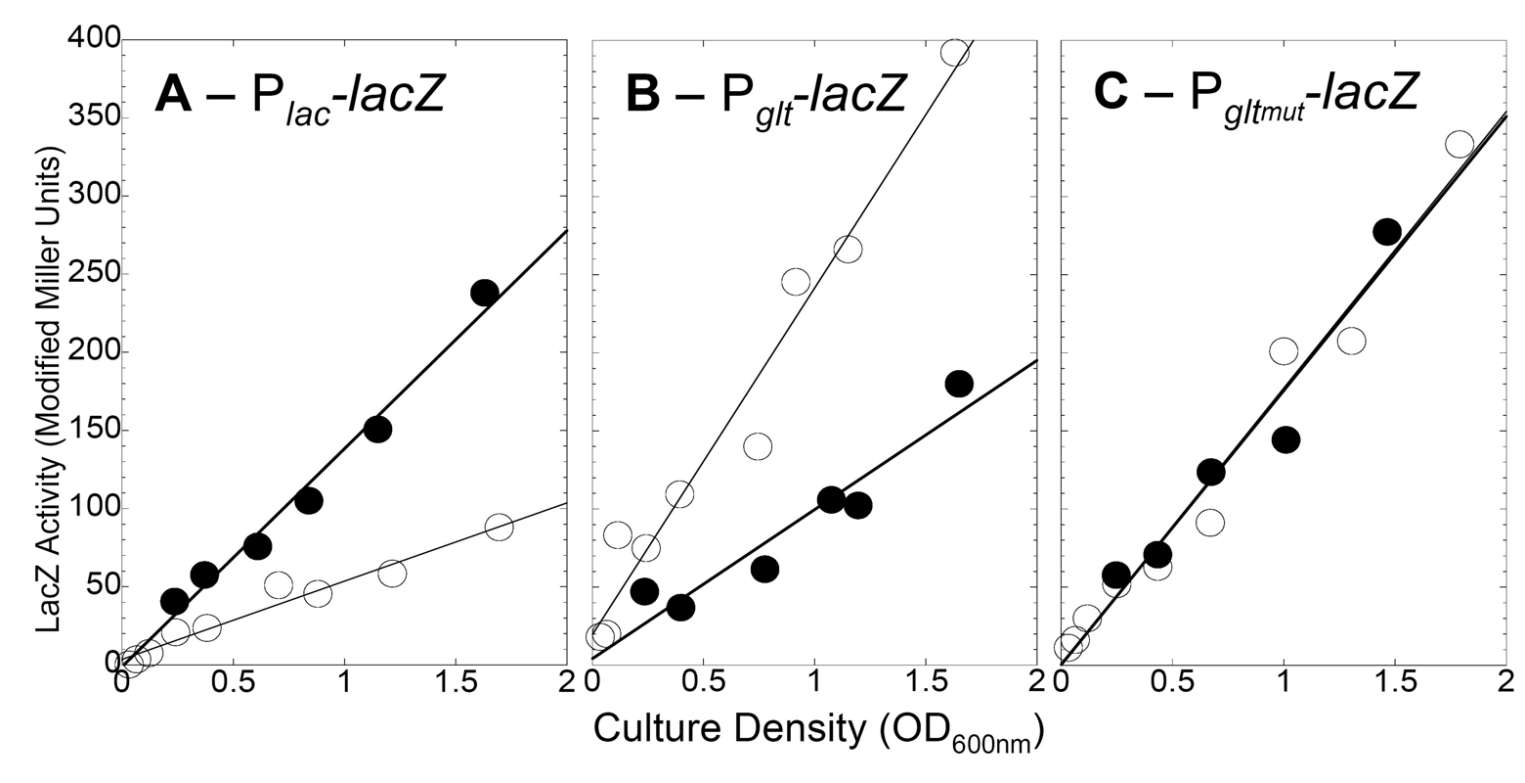
**Effects of dibutyryl-cAMP on expression from P*gltBDF *and a mutant form with altered Crp binding sites**. Cells were grown in a MOPS-based defined glucose rich medium with (closed symbols) or without (open symbols) 10 μM dibutyryl-cAMP. All strains were Crp^+^. **A**. Expression of the native *lac *operon (strain W3110). **B**. Expression of a P*gltBDF-lacZ *fusion (strain PM2003). **C**. Expression of a fusion of *lacZ *to the mutant version of P*gltBDF *shown in the bottom line of Fig. 5A (strain PM2004).

Third, we determined the effects of the membrane-permeable derivative dibutyryl-cAMP on expression of *gltB-lacZ *with and without alteration of the Crp binding sequence. The alterations are shown in Fig. 5A (bottom line), and replace all of the most highly-conserved nucleotides of known Crp-binding sequences (shaded) that were not already different in the native *gltBDF *promoter. Concentrations of dibutyryl-cAMP as high as 50 μM had no effect on the growth rate of the cultures (not shown), and we used 10 μM in the experiments described here. As a control, parental strain W3110 (with an intact *lac *operon) was grown in a defined glucose rich medium plus the inducer IPTG. As shown in Fig. 6A, dibutyryl-cAMP had the expected effect of increasing *lacZ *expression in the parental strain, due to relief of catabolite repression [22]. In contrast, Fig. 6B shows that dibutyryl-cAMP reduced expression of *lacZ *fused to WT P*gltB*, while Fig. 6C shows that dibutyryl-cAMP had no effect on *lacZ *fused to P*gltB *having the altered Crp binding sequence. Similar results were obtained in glucose minimal medium (not shown).

As Crp most often acts as a transcription activator, we explored some possible explanations for its repressive effect on P*gltB*. One possibility, given the proximity of the Crp and IHF binding sites (Fig. 2A), and the requirement of IHF for activation by Lrp [14], is that Crp interferes with IHF binding. However mobility shift analysis indicates that Crp and IHF bind independently of one another (Fig. 5D), though the ternary and binary complexes could not be resolved from one another well enough to completely rule out limited negative (or positive) cooperativity in their binding.

**Figure 7:**
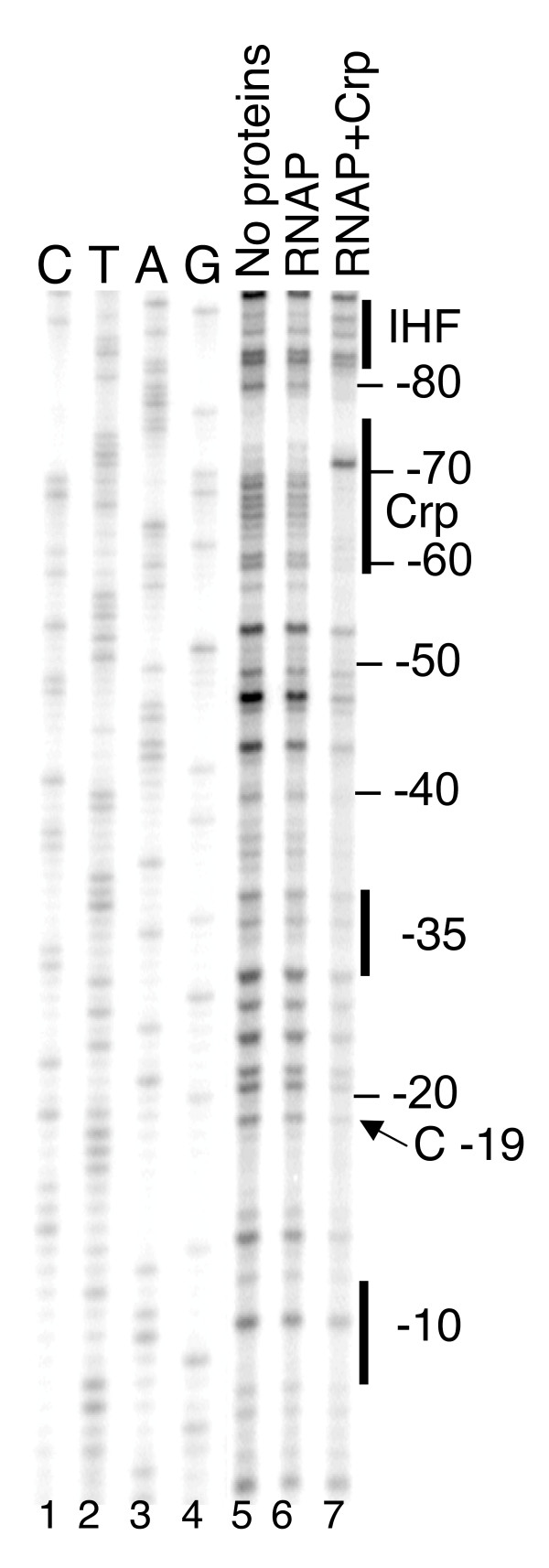
**Effect of Crp on protection of the *gltBDF *promoter by RNAP**. A fragment of *gltBDF *DNA from -571 to +51, labelled at the +51 end was used for DNase I protection assays. The first 4 lanes represent a sequencing ladder of the region. RNAP was used at 30 nM and Crp at 100 nM. Reactions with CRP contained 2mM cAMP.

A second possibility, which we did not test, is that the Crp-induced bend in the DNA [23] opposes that generated by IHF, preventing proper activation. A third possibility is that Crp interferes with RNAP binding. As noted above, RNAP alone fails to yield a clear protection pattern on P*gltB *DNA. Adding Crp-cAMP, far from interfering with RNAP binding, resulted in the appearance of a well-defined pattern of RNAP-dependent protection (Fig. 7). This pattern is, however, distinct from that seen when the coactivators Lrp and IHF are added in place of Crp (Fig. 3B). One particularly striking difference, as an example, is the DNase cleavage at position -19, that shows hypersensitivity in the presence of Lrp + IHF but not in the presence of Crp (compare lane 7 of Fig. 3B to lane 7 of Fig. 7).

### ArgR-Arg regulation of *gltBDF *transcription

The Ciphergen ProteinChip™ analysis referred to above yielded another *E. coli *polypeptide bound to P*gltB *DNA, having a molecular mass close to that of the regulatory protein ArgR (Fig. 4A). The involvement of ArgR in *gltBDF *regulation is reinforced by the following three observations. First, adding 7.5 mM L-arginine to MOPS-glucose medium nearly halved LacZ activity, in the *gltB-lacZ *fusion strain LP1000 (and its isogenic *rph*^+ ^strain LP2020; see below) (Table 1). Second, deleting *argR *from strains LP1000 (LP1050) or LP2020 (LP2023) doubled LacZ activity. Third, adding arginine to the growth medium did not affect LacZ levels in the Δ*argR *strain LP1050.

**Figure 8:**
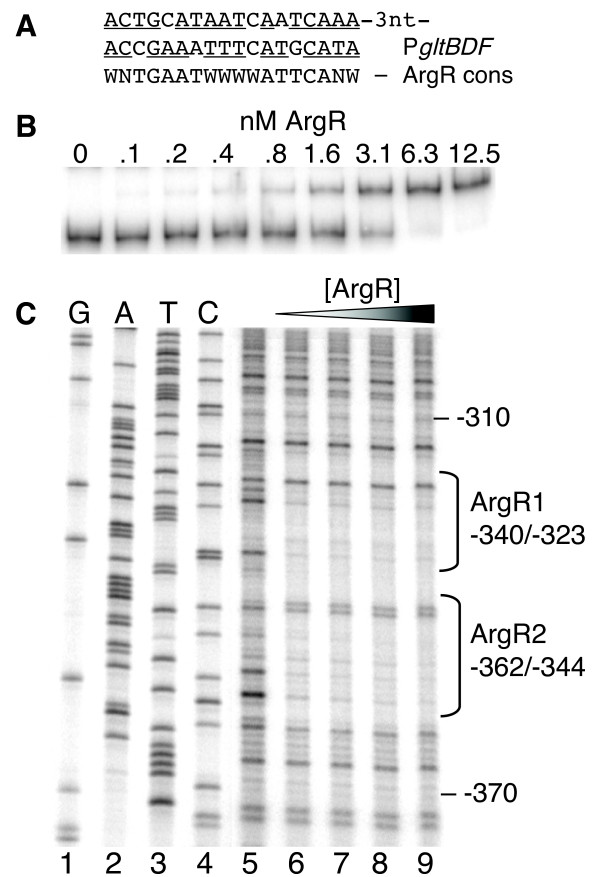
**Interactions between ArgR and the *gltBDF *promoter region**. **A**. The ArgR-binding sequences upstream of *gltBDF *(top two lines) compared to the symmetrical consensus ArgR-binding site [64]. Matches to the consensus are underlined. W = A or T, and N = A, C, G or T. **B**. Gel mobility shift analysis of ArgR binding to the region -406 to +246 of *glt *DNA. L-arginine was present in the reactions, the gel and running buffer (see Methods). **C**. DNase I protection assay of the region -462 to +161 bp upstream of *gltBDF*, labelled at the -462 end of the partner strand. The first 4 lanes represent a sequencing ladder of the partner strand in this region. ArgR concentrations of 0, 3.13, 6.25, 12.5, and 25 nM were used, and 5 mM L-arginine was present.

We have evidence that ArgR mediates its regulatory effect on the *gltBDF *promoter via direct interaction. There are two good matches to the consensus ArgR binding site sequence [24,25] upstream of the promoter-distal Lrp binding site (Figs. 2A and 8A). When *gltBDF *DNA containing the putative ArgR sites upstream of -270 was deleted, arginine had no effect on *gltB-lacZ *expression (strain LP1270, Table 1). We extended this observation by using purified ArgR in mobility shift and DNAse I footprint analyses. ArgR bound to *gltB *DNA (-406 to +246) with a *K*_d_^app ^of 1.8 ± 0.7 nM (Fig. 8B), and to a 3'-shortened fragment (-406 to -128) with essentially the same affinity (*K*_d_^app ^of 1.7 ± 0.06 nM; not shown). These data were fitted using the Hill equation as previously described [26-28], and both binding curves were characterized by a Hill coefficient of 2.0, indicating that multiple ArgR molecules are binding cooperatively.

The sequence to which ArgR binds was determined by DNase I protection using *gltB *DNA from -462 to +161. ArgR protected a region upstream of the distal Lrp binding site (Figs. 2A and 8C), extending from -361 to -318 on the non-template strand. This encompasses the two ArgR consensus-matching sites, and is consistent with the Hill coefficient of 2.0. Similar results were obtained for the template strand (not shown; a longer fragment, extending from -575 to +131, gave lower resolution due to the position of the footprint on the fragment).

**Figure 9:**
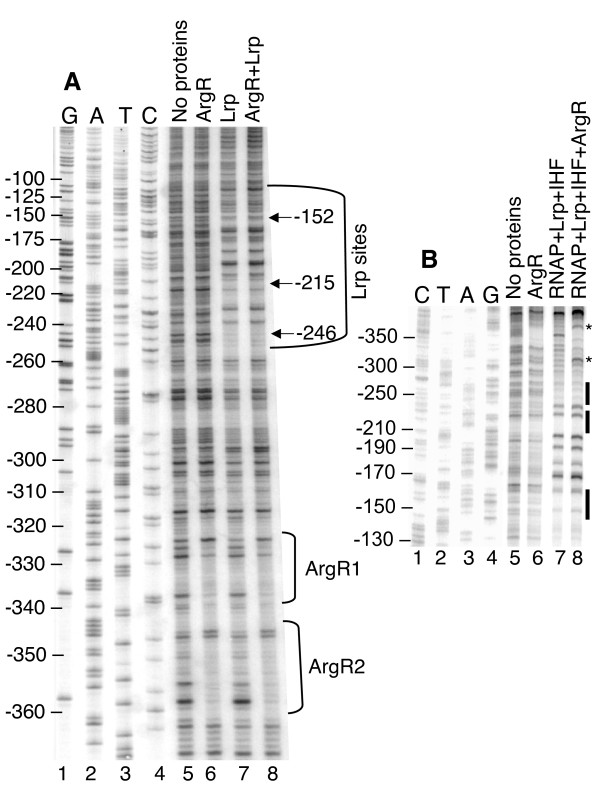
**Effect of ArgR on protection of the *gltBDF *promoter by RNAP and Lrp**. The first 4 lanes represent the sequencing ladder of the region. **A**. A *gltBDF *DNA fragment from -462 to +161 labeled at the -462 end was used. The concentrations of both Lrp and ArgR were 25 nM. **B**. A fragment of *gltBDF *DNA from -571 to +51, labeled at the +51 end was used. The concentrations of proteins used were as follows: ArgR 25 nM, RNAP 50 nM, Lrp 25 nM and IHF 50 nM. Reactions contained 5 mM L-arginine.

We considered two possibilities for the basis of *gltBDF *repression by ArgR. First, given the proximity of the ArgR and Lrp binding sites (Fig. 2A), we tested whether ArgR interfered with Lrp binding. Mobility shift assays with ArgR and Lrp were inconclusive in this respect, because adding the corepressor arginine to the gel prevented the Lrp-DNA complex from migrating far into the gel (not shown). However DNAse I footprint analyses suggest that Lrp and ArgR bind independently of one another (Fig. 9A).

A second possible basis for repression of *gltBDF *by ArgR involves interference with the proposed Lrp-RNAP interaction. ArgR does in fact alter the protection pattern generated by the combination of Lrp, IHF, and RNAP (Fig. 9B, asterisks). In the absence of ArgR there is a limited region of RNAP-dependent hypersensitivity that overlaps the ArgR binding site; addition of ArgR and arginine alters this hypersensitivity pattern.

### Effects of rph on *gltBDF *expression

We considered the possibility that our measurements of *gltBDF *expression were being affected by a defect in the *E. coli *W3110 background. This commonly-used *K-*12 strain carries a frameshift mutation in *rph*, the structural gene for RNAsePH, that results in decreased *pyrE *(orotate phosphoribosyltransferase) expression and in turn leads to pyrimidine limitation during growth in minimal media [29]. Pyrimidine biosynthesis is regulated by feedback inhibition, so it seemed plausible that W3110 would sense increased demand for glutamate (to provide needed glutamine precursors), and would thus have elevated *gltBDF *expression. Consistent with this possibility, adding 20 μg/ml of the pyrimidine uracil to strain LP1000 in minimal medium resulted in a 1.5 fold reduction of P*gltB-lacZ *activity (strain LP1000 is a W3110 derivative carrying a *gltB-lacZ *fusion in a Δ*lac *background). We replaced the W3110 *rph *allele with the wild-type gene (see Methods), and the new strain was designated LP2002. Correcting the *rph *mutation roughly halved transcription from P*gltB *in strain LP2020 (strain LP2002 bearing Δ*lac *and a *gltB-lacZ *fusion, see Table 2), in agreement with the uracil addition results. Strain LP2002 may be generally useful for studies of *E. coli *physiology.

## Discussion

### Role of IHF in mediating activation of *gltBD *by Lrp

IHF, a DNA-bending protein [30], is required for activation of *gltBDF *transcription by Lrp [14]. IHF subtly affects the Lrp-dependent DNAse I footprint (Fig. 3A), suggesting that IHF alters the Lrp-DNA complex (or alters DNAse I access to that complex). In addition, IHF bending could bring the Lrp-DNA complex into activating contact with RNAP at P*gltB *(Fig. 2B). IHF plays an architectural role at several activated promoters, such as P*ilvP *[31]. We have not directly demonstrated looping, but two results support this view. First, no defined footprint for RNAP was seen at P*gltB *with either IHF or Lrp alone, only with both together (Fig. 3B and our unpublished results). Second, an earlier study demonstrated that a 5 bp (half helical turn) insertion between the proximal Lrp binding site and the promoter reduced *gltB-lacZ *expression 3-7-fold, while a 10 bp (full helical turn) insertion at the same location did not reduce fusion expression (Fig. 5B of Wiese *et al*. [26]). Bending or looping models do not rule out the possibility of direct RNAP-IHF contacts occurring as well [32].

### Does Crp pre-position RNAP in response to energy metabolism?

The flux through the GlnA/GltBD pathway can be quite high in minimal media. ATP is required for the GlnA/GltBD pathway and not for the GdhA pathway (Fig. 1), and governing the relative fluxes through these alternative pathways is probably one of the most critical regulatory problems faced by *E. coli*. We report direct regulation of P*gltB *by Crp, in agreement with earlier indirect evidence [15]. A microarray-based global analysis of *E. coli *genes affected by Crp [33] did not identify the *gltBDF *operon, but this probably reflects the rich culture medium (LB) used in that work. Crp is broadly associated with the control of carbon and energy source utilization, usually activating genes for utilization of less-efficient carbon/energy sources (relative to glucose) [33]. Competitions between *gdhA *and *gdhA*^+ ^strains [3,4] reveal a clear advantage for the *gdhA*^+ ^strain during glucose limitation, suggesting that Crp modulates the relative activities of the two alternative pathways for glutamate synthesis to favor the ATP-independent pathway during growth on suboptimal carbon sources [3,4]. The reciprocal effects of carbon source and addition of cAMP on GdhA and GltBD activities are consistent with just this role for Crp [15]. In addition, Crp-cAMP represses one of the promoters for the glutamine synthetase gene (*glnAp2*) [34], so both enzymes for the GlnA/GltBD pathway are controlled in parallel by Crp.

Our data show that Crp-cAMP binding *in vitro *(Fig. 5B, E) is correlated with reduced transcription *in vivo *(Fig. 6), and that RNAP generates a protection pattern at P*gltB *in the presence of Crp-cAMP (Fig. 7, lane 7) different from the pattern yielded by RNAP in the presence of IHF + Lrp (Fig. 3B, lane 7). The Crp-cAMP repression thus appears to alter RNAP binding rather than blocking it. The Crp footprint is centered at -65.5 relative to the start of *gltB *transcription, close to the position from which Crp activates class I promoters [35] where Crp binds between the C-terminal domain of RpoA (αCTD) and region 4 of RpoD (σ^70^). However optimal spacing from the proximal edge of the Crp binding site to that of the -35 hexamer is 13–16 bp in class I promoters, while in P*gltB *this spacing is 21 bp (Fig. 2A). Crp may thus repress P*gltB *by mispositioning or trapping RNAP. Repression has been seen due to mispositioning of RNAP via alternative binding sites [36], or trapping at the promoter [37,38]. A recent global analysis suggests that nearly a quarter of σ^70 ^(RpoD)-dependent promoters have bound RNAP that is "poised" but not transcribing [39]. RNAP poised at P*gltB *would allow a rapid increase of GltBD levels in the face of falling ammonium concentrations, as the GdhA reaction becomes increasingly inefficient (Fig. 1).

### Role of ArgR in repressing *gltBDF*

As shown in Fig. 1, glutamate is a precursor of ornithine, which in turn is converted to arginine. Thus regulation of GltBD in response to arginine is an example of negative feedback regulation. The relatively modest effects are appropriate given the need for glutamate in many other pathways. A similar feedback regulation of glutamate biosynthesis by ArgR was recently observed in *Pseudomonas aeruginosa *[40]. The mechanism by which ArgR represses P*gltB *is not clear, but our evidence is consistent with the possibility that ArgR interferes with formation of the final activation complex. As shown in Fig. 8, and illustrated in Fig. 2, ArgR binds far upstream of the promoter, near the Lrp-binding region. However ArgR does not appear to have a major effect on Lrp binding (Fig. 9A). Our working hypothesis is that ArgR allows pre-assembly of the Lrp-IHF-RNAP complex, but blocks a specific contact required for transcriptional activation. This would resemble the suggested role of Crp (see above) in reducing *gltBD *expression while allowing rapid return to full activation should the appropriate conditions change.

### Role of Lrp in regulation of *gltBD*

Lrp is not simply a signal of amino acid sufficiency. First, *gltBDF *is one of the operons for which Lrp regulation is relatively insensitive to the coregulator leucine [5,28]. Second, Lrp levels (like those of the coactivator IHF [41]) are sensitive to the alarmone ppGpp [42], which in turn is affected by sufficiency of both amino acids and other nutrients [43]. Lrp levels vary between media, and (like those of the coactivator IHF [41]) also through the growth cycle [42].

Much of what is known about regulation of GdhA, GlnA, and GltBD is consistent with the expected reciprocal pattern between the two pathways shown in Fig. 1. One would expect GdhA levels to parallel those of [NH_4_^+^], while GlnA and GltBD levels would perhaps follow the inverse of [NH_4_^+^] and parallel those of ATP. In fact, *gdhA *is repressed by Nac, with repression relieved in high [NH_4_^+^], while neither *gln*A nor *gltBD *appear to respond substantially to Nac [17,44,45]. Similarly, transcription of both *glnA *and *gltBD *is repressed by Crp-cAMP while effects on *gdhA *are unclear (this work, [34,46]). Conversely, both GlnA activity (indirectly, via *glnL*) and *gltBD *transcription are boosted by Lrp, which has no obvious effect on *gdhA *[5,6,28].

Modeling analysis [47] suggests that the major control of relative flux between the alternative pathways shown in Fig. 1 should involve regulation of GlnA activity (via adenylylation and deadenylylation [48]). Lrp has major effects on GlnA expression and adenylylation status in response to ammonia levels [2,5,7]. The effect of Lrp on GlnA expression and activity is indirect, probably involving the direct Lrp effect on GltBD expression that, in turn, modulates the intracellular ratios of α-ketoglutarate, glutamate, and glutamine [49]. This model requires changes in gene expression levels for adaptation to sudden large changes in [NH_4_^+^], with *gltBD *needing to be expressed above a threshold level. The 30-40-fold activation of P*gltB *by Lrp [28] is consistent with a major switch above or below this critical threshold expression level. In contrast, the effects of Crp and ArgR (and of leucine, in this case) are smaller, fine-tuning controls that may modulate the flow of glutamine out of the middle of the GlnA-GltBD pathway.

## Conclusion

Results from this study demonstrate that the physiologically-important *gltBDF *operon of *E. coli *is subject to negative as well as positive control, and involves a third member of the group of seven most-global transcriptional regulatory proteins. In addition to the previously-demonstrated direct positive roles of Lrp and IHF, there are direct negative roles for Crp and ArgR. Lrp and IHF appear to stabilize RNAP binding to the promoter, as no RNAP-dependent footprint appears unless both Lrp and IHF are present. Crp-cAMP can replace Lrp + IHF in potentiating footprint formation by RNAP, though the resulting protection patterns are distinct. ArgR alters, but does not prevent, footprint formation by RNAP + Lrp + IHF. It does not appear that either repressor, Crp or ArgR, acts by interfering with binding of the activators or of RNAP. This study does not explore the specific mechanisms of activation or repression at *gltBDF*, but the results are consistent with the possibility that Crp and ArgR inhibit transcription in such a way as to leave the RNAP poised for rapid transcription initiation when conditions require it. Given the central importance of the regulation of glutamate biosynthesis, reflected by the involvement of Lrp and two other *E. coli *global regulatory proteins, it would be instructive to know whether this regulatory mechanism is broadly conserved among related bacterial species adapted to different nutritional environments.

## Methods

### Bacterial strains, plasmids and culture conditions

The strains, plasmids and PCR primers used in this study are listed in Table 2. All strains were derivatives of *E. coli K-*12 W3110 or LP2002 (in which the *rph *mutation of W3110 was corrected; see below). For β-galactosidase assays, strains were grown in glucose minimal MOPS medium [50,51] containing ampicillin (20 μg/ml for chromosomal and 80 μg/ml for plasmid-borne resistance) or 7.5 mM L-arginine HCl (Sigma) where indicated. Cultures grown to test the effects of Crp-binding site mutations were grown in MOPS defined-rich medium (Teknova, Hollister, CA) containing 100 μg ampicillin per ml and 1 mM isopropyl-β-D-thiogalactoside (IPTG), in the presence or absence of 10 μM dibutyryl-cAMP (BioMol Research Labs, Plymouth Meeting, PA). Plates for most genetic work used agar containing "LB" medium [52].

### Construction of the *rph*^+ ^strain LP2002 and its derivatives

The widely used *E. coli K-*12 strain W3110 contains a frameshift mutation in *rph *associated with decreased levels of the *pyrE *product orotate phosphoribosyltransferase [29]. This frameshift was corrected as follows. A 790 bp fragment of *rph *and downstream sequence was amplified from W3110 chromosomal DNA using the polymerase chain reaction (PCR) and the overlap extension procedure [53]. The primers used were rph1-4 (Table 2). Primers rph2 and rph3 contain the additional base pair required to correct the frame shift in the *rph *gene (underlined italic in Table 2). The PCR product was cloned into *E. coli *strain XL-1Blue (Stratagene) using the pGEMT-easy vector (Promega). The insert was then excised with NotI and ligated into NotI-digested suicide vector pKO3 [54]. Electrocompetent W3110 cells were transformed with pKO3 carrying the modified *rph *gene, and integrants were selected at 43°C. Cells that had lost the plasmid were isolated by plating on LB agar containing 5% sucrose, which selects against the plasmid *sacB *gene [54]. Colonies from LB-sucrose plates were replica plated onto unsupplemented LB and LB-chloramphenicol (20 μg/ml). Colonies that grew on LB plates but not on LB-chloramphenicol were screened for correction of the frame shift in the *rph *gene by amplifying the *rph *region by PCR and sequencing the product. The resulting *rph*^+ ^strain was designated LP2002.

The *lac *operon was deleted from strain LP2002 using the overlap extension protocol [53] with primers lac6-9 (Table 2). The lac6 primer corresponds to nucleotides 814 to 842 within the coding region of *lacI*; the lac7 primer is complementary to the 5' region of primer lac8; the 3' end of lac8 bridges the deletion region (see below), and lac9 corresponds to nucleotides 1019 to 1044 of the coding region of *cynX *gene downstream of the *lacZYA *operon. The final amplified product has a deletion starting immediately upstream of the -10 region of the *lacZYA *operon and extending to the 3' end of the *lacA *gene, leaving the last 19 codons of *lacA *intact. This product was introduced into the chromosome of LP2002 using the suicide vector pKO3 [54]. The Δ*lacZYA *recombinants were identified as white colonies on LB-Xgal plates, and the deletion was verified by amplifying the chromosomal *lac *region and sequencing the fragment. The resultant strain was designated LP2010. The *gltB-lacZ *transcriptional fusion from LP1000 [14,55] was introduced into LP2010 via P1 transduction [55] and designated LP2020.

### Construction of Δ*argR *strains

A portion of *argR *was deleted from strains LP1000 and LP2020 as follows. The *argR *gene from strain PS2209 (W3110 Δ*lac*-169 from F. C. Neidhardt) was amplified from chromosomal DNA by PCR, using the primers arg1 and arg2 (Table 2). The amplified product was ligated into the pGEMTeasy vector (Promega). The *argR *fragment was excised from the vector with NotI, and the region of *argR *coding for amino acids 15–90 were deleted in-frame by removing a DraI/EcoRV fragment from the *argR *gene and religating the blunt ends. This fragment was ligated into suicide vector pKO3 [54] and introduced into strains LP1000 and LP2020. Prospective mutants were screened by amplifying the *argR *region from their genomic DNA; the amplified fragment was shorter in the deletion mutants. The Δ*argR *strains were designated LP1050 (LP1000 derivative) and LP2023 (LP2020 derivative).

### CipherGen ProteinChip™ assays

Surface-enhanced laser desorption and ionization (SELDI) ProteinChip technology (Ciphergen Biosystems, currently distributed by BioRad, Hercules, CA) was used to identify proteins binding to the upstream region of the gltBDF operon. In this approach, biotin-labeled gltB DNA (from -406 to +246) was attached to streptavidin-coated chips and incubated with centrifugally-cleared whole-cell extracts. The masses of proteins that bound to the DNA fragments were determined using SELDI mass spectroscopy. This binding experiment was carried out under nonstringent conditions so as to detect relatively weak binding interactions. *E. coli *cells (strain W3110) were grown in glucose minimal MOPS medium and opened using glass beads in a BeadBeater (BioSpec Products, Bartlesville, OK). PS-1 (carbonyl diimidazole) ProteinChips were coated with streptavidin (Pierce, Immunopure™) at 0.2 microgram/spot, washed, and the unbound sites were blocked with 1 M ethanolamine (pH 8.0). The DNA, generated by PCR using biotinylated primers, was first bound to the streptavidin on the chip, and then incubated with cell extract in 20 mM Tris-acetate (pH 8.0), 0.1 mM EDTA, 0.1 mM dithiothreitol, 250 mM NaCl, 4 mM Mg acetate, 12.5% glycerol (v:v), and 200 μg/ml of salmon sperm DNA (Amersham Pharmacia Biotech, N.J.). The chips were washed with 20 mM Tris-acetate containing 0.1% Triton X-100. The matrix EAM-1 (Ciphergen) was added to the chip prior to SELDI mass analysis.

### Expression and purification of Lrp, ArgR, Crp and IHF

In all cases, protein purity was assessed from single (or, in the case of IHF, double) Coomassie-stained bands when μg amounts were loaded onto SDS polyacrylamide gels. Lrp was purified from strain JWD3 and isolated as previously described [28]. The genes *argR*, *crp*, *himA *and *himD *(the latter two coding for IHF subunits) were amplified from strain W3110 chromosomal DNA. The *argR *gene was amplified using primers arg3 and arg4 (Table 2), cloned into the expression vector pET23b (Novagen) at the *Nde*I/*Xho*I sites creating pLP1060, and introduced into the expression host BL21(DE3) (Novagen) which carries an inducible gene for T7 RNAP. The protein was purified from the resulting strain LP1060, as described by Sunnerhagen *et al*. [56].

The *crp *gene was amplified using primers crp5 and crp6 (Table 2), ligated into the *Nde*I/*Xho*I sites of expression vector pET29a (Novagen) to create pLP1070, and introduced into strain BL21(DE3) resulting in strain LP1070. The overexpressed protein was purified using a nickel affinity column (Pharmacia) since the wild type Crp (without a his-tag) binds nickel columns with moderate affinity [57].

The genes *himA *and *himD *were amplified from W3110 chromosomal DNA using primers himA1 and himA2 and primers himD1 and himD2 (Table 2). The amplified products were ligated in tandem into the expression vector pET28a (Novagen) at the *Nco*I/*Bam*HI sites to create pLP1080, and introduced into strain BL21(DE3). The strain was designated LP1080. IHF with an N-terminal histidine tag on HimA was purified using a 3 ml Hi-Trap Heparin Sepharose column from Pharmacia [58].

### Construction of P*gltB-lacZ *fusion and variant with altered Crp-binding site

The promoter region upstream of *gltB *was amplified from *E. coli *chromosomal DNA using primers gltP1-2 (Table 2), and the resulting PCR product was digested with BamHI and SalI. This product was ligated into pBH403, a derivative of pKK232-8 with a promoterless *lacZ *gene between two bidirectional transcription terminators, to generate pPM2005 (Table 2). This process was repeated with a mutagenic primer pair (gltcrp1-2, Table 2) to generate pPM2006, which has alterations within the Crp-binding site in the *glt *promoter. Plasmids were sequence confirmed and used to transform the Δ*lacZ *W3110 derivative PS2209.

### β-galactosidase assays

Cultures were grown to exponential phase in the indicated media. Samples were taken at 30-min intervals throughout the growth period. Levels of β-galactosidase were determined by *o*-nitrophenyl-β-D-galactoside (ONPG) hydrolysis as described by Platko *et al*. [59]. β-galactosidase levels were plotted against culture absorbance, and points were fitted via linear regression. The resulting slope yields the β-galactosidase acitivity.

### Mobility shift and DNase I protection assays

RNA polymerase holoenzyme was purchased from Epicentre Biotechnologies (Madison, WI). Assays were carried out as described in Paul *et al*. [14] except where indicated. In electrophoretic mobility shift experiments involving ArgR, the reaction mixture and the polyacrylamide gel both contained 5 mM L-arginine, and the running buffer (1 × TBE [90 mM Tris borate, pH 8.3, 0.2 mM EDTA]) contained 1 mM L-arginine. Crp-DNA binding was carried out in a buffer modified from Seoh and Tai [60] containing 10 mM Tris-HCl (pH 8.0), 50 mM KCl, 1.0 mM EDTA, 0.1 mM DTT, 50 μg/ml BSA, 10 μg/ml poly (dI-dC):poly(dI-dC) from Pharmacia and 12.5% glycerol. cAMP (20 mM, Sigma) was added to the reaction buffer, the gel and the running buffer (0.5 × TBE). The buffer for DNase I protection assays (10 mM Tris-HCl, pH 8.0, 5 mM MgCl_2_, 1 mM CaCl_2_, 2 mM dithiothreitol, 50 mg of bovine serum albumin/ml and 2 μg of poly(dI-dC):poly(dI-dC)/ml) contained 5 mM L-arginine or 20 mM cAMP for reactions involving ArgR and Crp respectively.

## Authors' contributions

LP carried out most of the laboratory experiments, with PM responsible for those involving alteration of the Crp binding sites and tests of their function. LP, RB and RM planned the experiments and analyzed the data. All authors contributed to the writing of the manuscript, with RB having primary responsibility for the writing and the figures. All authors read and approved the final manuscript.

## Figures and Tables

**Table 1:** Effects of arginine and ArgR on *gltB*::*lacZ *expression

**Effect tested**	**Strain/condition**	**LacZ activity (units)**^1^	**Normalized data**
	LP1000 (*rph*)	1172^2^	(1.00)

Addition of arginine^3^	LP1000 + Arg	660	0.56
	LP1270	900	0.77
	LP1270 + Arg	953	0.81
	LP2020 (*rph*^+^)	736	0.63
	LP2020 + Arg	402	0.34

Deletion of ArgR	LP1050 (Δ*argR*)	2981	2.54
	LP1050 + Arg	3035	2.59
	LP2020 (*rph*^+^)^4^	648	0.55
	LP2023 (*rph*^+ ^Δ*argR*)	1283	1.09

^1 ^One unit of LacZ (β-galactosidase) activity is defined as 1 nmol of ONPG hydrolyzed per min.

^2 ^Samples were taken at several culture densities, and the resulting LacZ activity was plotted *vs*. culture density (as in Fig. 6). The activity was calculated from the slope of the line; in every case, the correlation coefficient of the least squares regression was ≥ 0.99.

^3 ^Arginine hydrochloride was added to the medium to a final concentration of 7.5 mM.

^4 ^These results were obtained on a different day from the other experiments.

**Table 2:** *E. coli *strains^1^, plasmids, and primers used in this work

Strain/plasmid/primer	Description	Source or reference
**Strains**		
BE3471	PS2209 *gltB (psiQ39*)::*lacZ *(Mu d1-1734)	[28]
BE3479	PS2209 *gltB (psiQ32*)::*lacZ *(Mu d1-1734)	[28]
BE3779	PS2209 *gltB (psiQ32*)::*lacZ *(Mu d1-1734) *lrp-35*::Tn*10*	[28]
BL21(DE3)	F^- ^*ompT hsdS*_*B *_(r_B_^-^m_B_^-^) *gal dcm *(DE3)	Novagen
JWD3	BE1 (W3110 *lrp-201::*Tn*10*) pJWD2	[28]
LP1000	PS2209 *gltB*::*lacZ *transcriptional fusion at λ_att _with -406 to +246 of the *gltBDF *operon	[14]
LP1050	LP1000 Δ*argR*	This work
LP1060	BL21DE3/pLP1060	This work
LP1070	BL21DE3/pLP1070	This work
LP1080	BL21DE3/pLP1080	This work
LP1270	PS2209 *gltB*::*lacZ *transcriptional fusion at λ_att _with -270 to +246 of the *gltBDF *operon	This work
LP2002	W3110 *rph*^+^	This work
LP2010	LP2002 Δ*lacZYA*	This work
LP2020	LP2010 *gltB*::*lacZ *transcriptional fusion at λ_att _with -406 to +246 of the *gltBDF *operon	This work
LP2023	LP2020 Δ*argR*	This work
PM2003	PS2209/pPM2005	This work
PM2004	PS2209/pPM2006	This work
PS2209	W3110 Δ*lac-169*	F. C. Neidhardt
W3110	F^- ^prototroph *rph*	F. C. Neidhardt
XL1-Blue	*recA1 endA1 gyrA96 thi-1 hsdR17 supE44 relA1 Δlac-pro *[F*' proAB lacI*^q^*ZΔM15 *Tn*10*]	Stratagene
**Plasmids**		
pBH403	pKK232-8 with promoterless *lacZ *gene inserted upstream of *cat *gene in a derivative of pBR322	[61, 62]
pPM2005	pBH403 with *gltB *promoter region cloned from BamHI to SalI	This work
pPM2006	pPM2005 with mutations in the Crp-binding sequence	This work
PJWD2	p*Trc*99A (Pharmacia) with the *lrp *coding sequence cloned into the NcoI site	[28]
pLP1060	pET23b (Novagen) with the *argR *coding sequence cloned into the NdeI/XhoI sites	This work
pLP1070	pET29a (Novagen) with the *crp *coding sequence inserted into the NdeI/XhoI sites	This work
pLP1080	pET28A (Novagen) with the *himA *and *himD *coding sequences inserted in tandem into the NcoI//BamHI sites	This work
pGEMT-easy	Cloning vector	Promega
pKO3	Suicide vector	[54]

**Primers**		
arg1	CGCCAGCAGCGCCGAGGACTGCGAC	
arg2	CACTAATTATTGAGCTAATTAATACCGCGC	
arg3	CATATGCGAAGCTCGGCTAAG	
arg4	CTCGAGTTATTAAAGCTCCTGGTCGAACAGCTC	
crp5	CCATATGGTGCTTGGCAAACCGC	
crp6	CCTCGAGTTAACGAGTGCCGTAAACGAC	
gltP1	CGGGATCCCATAATCACATAAATCACTTTTGCTTATC	
gltP2	ACGCGTCGACAGCGGATTTCCAACTTATCG	
gltcrp1^2^	CAGTCAATTAATAAAGAATATAACGCTAAAGGCGGTTTCTGTACCAATAAGCTTGCC	
gltcrp2	GGCAAGCTTATTGGTACAGAAACCGCCTTTAGCGTTATATTCTTTATTAATTGACTG	
himA1	GGTACCGGCATCATTGAGGGATTGAACCTATGGCGCTTACA	
himA2	CTCGAGCTCGTCTTTGGGCGAAGC	
himD1	GGTACCTTAACCGTAAATATTGGCGCGATCGCG	
himD2	TCATGACCAAGTCAGAATTGATAGAAGAC	
lac6	GGATACGACGATACCGAAGACAGCTCATG	
lac7	CGAGCCGGAAGCATAAAGTGTAAAGC	
lac8	GCTTTACACTTTATGCTTCCGGCTCGGGCGTTCCTTGTCGGGTTATTCG	
rph1	GCGTCTGATCGCCCGTGCTCTTCGCGC	
rph2^3^	CGTCGCTACAATGGATTCGATTCCCC*C*TCGGGC	
rph3^3^	GCCCGA*G*GGGGAATCGAATCCATTGTAGCGACG	
rph4	ACGGCAGGTCCAGGTCGTGATGCTCCG	

^1^All strains are *Escherichia coli K*-12.

^2^The underlined bases alter the Crp-binding site in the *gltBDF *promoter.

^3^The underlined italic base is inserted to replace the base deleted in the *rph *mutation.
